# Identifying bereaved subjects at risk of complicated grief: Predictive value of questionnaire items in a cohort study

**DOI:** 10.1186/1472-684X-10-9

**Published:** 2011-05-16

**Authors:** Mai-Britt Guldin, Maja O'Connor, Ineta Sokolowski, Anders B Jensen, Peter Vedsted

**Affiliations:** 1 Research Unit for General Practice, Aarhus University, Aarhus, Denmark; 2 Psychooncological Research Unit, Aarhus University, Aarhus, Denmark; 3 Department of Oncology, Aarhus University Hospital, Aarhus, Denmark

## Abstract

**Background:**

Bereavement is a condition which most people experience several times during their lives. A small but noteworthy proportion of bereaved individuals experience a syndrome of prolonged psychological distress in relation to bereavement. The aim of the study was to develop a clinical tool to identify bereaved individuals who had a prognosis of complicated grief and to propose a model for a screening tool to identify those at risk of complicated grief applicable among bereaved patients in general practice and palliative care.

**Methods:**

We examined the responses of 276 newly bereaved individuals to a variety of standardised and ad hoc questionnaire items eight weeks post loss. Inventory of Complicated Grief (ICG-R) was used as a gold standard of distress at six months after bereavement. Receiver operating characteristic (ROC) curves analysis was performed for all scales and items regarding ICG-R score. Sensitivity, specificity and area under curve (AUC) were calculated for scales and items with the most promising ROC curve analyses.

**Results:**

Beck's Depression Inventory (BDI) was the scale with the highest AUC (0.83) and adding a single item question ('Even while my relative was dying, I felt a sense of purpose in my life') gave a sensitivity of 80% and specificity of 75%. The positive/negative predictive values for this combination of questions were 70% and 85%, respectively. With this screening tool bereaved people could be categorized into three groups where group 1 had 7%, group 2 had 23% and group 3 had 64% propensity of suffering from complicated grief six months post loss.

**Conclusions:**

This study shows that the BDI in combination with a single item question eight weeks post loss may be used for clinical screening for risk of developing complicated grief after six months. The feasibility and clinical implications of the screening tool has to be tested in a clinical setting.

## Background

Bereavement is an existential condition experienced at some time in life by most people. Most individuals adjust adequately to the loss of a relative, nevertheless, a small but noteworthy proportion of bereaved individuals experience a syndrome of prolonged psychological distress in relation to bereavement. Prolonged distress and disability in connection with bereavement has been termed complicated grief (CG) or *Prolonged Grief Disorder *(PGD) [1].

PGD has been proposed for a new diagnosis in the DSM-V and has been shown to be a disorder distinct from Posttraumatic Stress Disorder (PTSD), depression and anxiety [1]. Factor analytic studies have supported items on a complicated grief reactions scale as separable from depression and anxiety [2]. Other studies indicate a significant overlap between symptoms of CG and PTSD [3]; [4], yet despite the unresolved issues on CG or PGD as a diagnostic entity, it has been noted, that complications during a time of grief is a debilitating condition in need of treatment [5]. So far there seems to be consensus on a diagnosis labelled PGD [1]. Left untreated CG has been shown to be associated with increased medicine consumption, problems with job retention, development of psychopathological disorders and increased mortality [6]; [1,7]. A recent longitudinal study using psychiatric interviews indicates that the prevalence of PGD or CG may be around 11% among bereaved individuals losing a close relative [8]. The focus of the present study is on complicated grief reactions and therefore, while keeping in mind the potential overlap between CG and PTSD [4], CG was chosen above PTSD or other syndromes as the outcome measure of bereavement related distress in this study.

Symptoms of CG have in numerous studies been assessed with the rating scale, *Inventory of Complicated Grief *(ICG) [1,5,9]. Items on the ICG correspond closely to the symptoms in CG and the proposed diagnosis of PGD. According to the consensus diagnosis, PGD or CG cannot be diagnosed until six months post loss. Accurately and early identifying persons at risk of developing CG would be advantageous in providing appropriate support as well as evidence-based treatment in primary and palliative care [10,11]. A major challenge for clinicians consists in correctly identifying vulnerable individuals susceptible to develop CG among the group of bereaved individuals [12]. A number of risk factors have been identified, such as attachment style, lack of social support and sudden loss [13,14]. Thus, there is a need for a clinical tool that can reliably assess the risk of developing CG in newly bereaved people. The aim of this study was to develop a clinical tool to identify bereaved individuals to establish a prognosis of CG at six months post loss and to propose a model for a screening tool for early identification of bereaved individuals at risk of CG applicable in general practice and palliative care.

## Methods

### Setting and procedure

The study was approved by the regional ethics committee and the study population consisted of two samples. One sample was a longitudinal cohort with measurement at 2, 6, 13 and 18 months (T1, T2, T3 and T4 respectively) post loss using a self-report questionnaire sent by mail. At T1 the questionnaire was administered through structured interviews at home visits to half of this sample. Postal questionnaires were used by all participants at T2-T4. This sample was identified via the Danish Central Person Register (CPR) and consisted of all persons aged 65 - 80 in the former county of Aarhus, Denmark, who had lost a spouse during the year of 2006 [4]. The Danish CPR contains personal information regarding age, marital status, name of partner and place of residence. The second sample was recruited via the palliative home care team at Aarhus University Hospital, Denmark. All relatives to newly deceased patients with whom the palliative home care team was in contact in the year of 2006 were asked to participate. This study population was contacted by mail approximately eight weeks post loss with an invitation to participate in the study along with a self-report questionnaire. Respectful reminders were sent by mail to non-responders after two weeks.

### Participants

**Figure 1:**
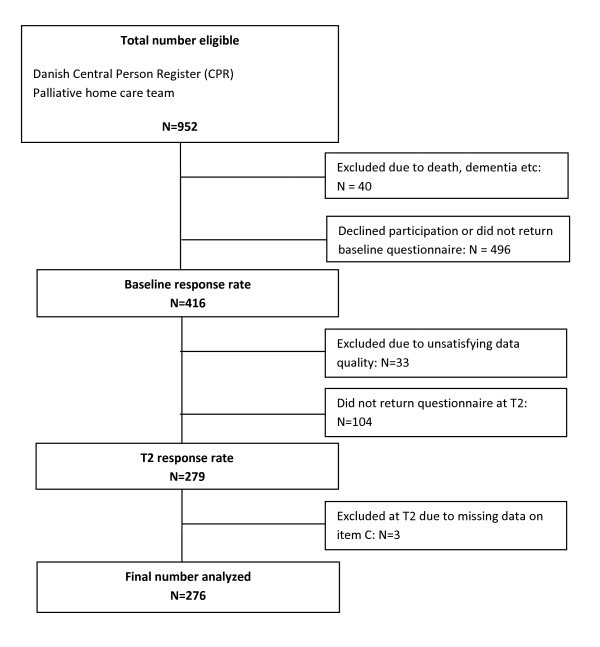
**Flowchart of participants analyzed in the study**.

Nine hundred fifty two bereaved were contacted, 838 sampled through the CPR and 114 via the palliative home care team (see Figure 1). Forty (40) individuals were excluded from the study due to reasons such as death, hospitalisation and dementia. Four hundred sixteen individuals (46%) agreed to participate in the study. Thirty-three (33) cases were excluded from the analyses due to more than 15% missing items. Thus, at baseline (T1), 383 of the eligible bereaved people participated in the study. Due to non-response of 104 participants at the first follow-up (T2) data from 276 participants was analysed for this study.

### Measures

Data collection was based on self-report questionnaires. Based on findings in earlier studies on CG, we wanted to investigate depression, PTSD, coping style, social support and personality variables as possible risk factors [6,13]. The questionnaire contained the following standardized scales and single items: Inventory of Complicated Grief-Revised (ICG-R) [6]; The Beck Depression Inventory (BDI)[15]; The Harvard Trauma Questionnaire-Part IV (HTQ-16)[16]; The Crisis Support Scale (CSS) [17]; Coping Style Questionnaire (CSQ) [18]; Sense of Coherence (SOC) [19]; Satisfaction with Life Scale (SWLS) [20]; The NEO Personality Inventory (NEO-PI-R)[21]. The baseline questionnaire also contained socio-demographic questions on education, years of marriage and number of children and a number of single items on distress and meaning of life (see later).

### Inventory of complicated Grief-Revised (ICG-R)

In this study CG was measured with the ICG-R. The ICG-R is a modified and shorter version of the original Inventory of Complicated Grief (ICG), which consisted of 19 items [6]. The ICG was developed to assess maladaptive symptoms of loss and contains all symptoms proposed for the PGD diagnosis [7}. The ICG-R is based on 15 questions with a 5-point Likert-scale, a functional criterion and a duration criterion of six months. Due to the duration criterion of six months, the ICG-R was administered at six months as the earliest measuring point. The version of the ICG-R administered in this study, had been used in an earlier Danish study, where it had proven highly reliable with Cronbach's α>0.94; and a mean inter-item correlation of 0.52 [22]. The results of the ICG-R served as the gold standard in this study. We used the cut off point of the 20% most distressed based on a syndromal level as initially suggested by the authors of the scale [6]. Using this method the cut off point in the Danish population was set to an ICG-R score of 43 and above. Fifty four (19.6%) of 276 analyzed newly bereaved people were suffering from CG six months post loss according to this criteria.

### The Beck Depression Inventory

The Beck Depression Inventory (BDI) is a widely used 21-item self-report measure, which assesses cognitive/affective and somatic symptoms of depression [15]. The scale is based on statements rated by the respondent (range 0-3) according to the intensity experienced during the past two weeks. Due to ethical reasons item 21, which pertains to sexuality, was omitted from the scale in this questionnaire as the respondents had just suffered spousal loss and pilot testing showed that the question was considered offensive by the respondents. Depression rates were not calculated in this study and the omission of item 21 did not pose a problem to the analyses.

### Single items

The questionnaire contained three Likert-type single item questions on distress and meaning experienced in relation to the death of the relative. These questions were inspired by the literature on risk factors. The Likert-scale ranged from 1-7 (1 = *not at all *and 7 = *a lot*). The cut point for these questions was set to five or more based on a symptom criterion. The questions were: *A*. *How much distress did you experience in relation to your relative dying? B. Even in times of hardship, like while my relative was dying, I feel a sense of meaning in my life? C. Even while my relative was dying, I felt a sense of purpose in my life?*

### Data Analysis

Data analysis was performed using STATA 10.1. Answers at T1 were analyzed to explore their association with answers on the ICG-R at T2, six months post loss. Six months post loss was considered a relevant point in time for analysis in a clinical setting within primary care to ensure early detection, and for the sake of simplicity analysis of data at 13 and 18 months post loss were left out of this study. Expectation Maximization algorithm was performed to estimate missing answers on subscales with less than 15% missing answers to allow the calculation of total scores [23]. Scores for the single items B and C were reversed in the process of data analysis so a higher score denominated more distress. Receiver operating characteristic (ROC) curve analysis was performed for all scales and items on the data set and measured against the scores on ICG at six months post loss.

ROC curves plot sensitivity (true positive ratio) by 1-specificity (true negative ratio) for a series of cut off points established by the scale or responses to the single items [24]. The area under the ROC curve (AUC) represents an overall measurement of performance of the test, with 1.0 as a perfect test and 0.5 representing a test with no discriminating capacity. Only scales and items with an AUC > 0.65 were selected for further analysis. The "optimal" cut off points for the scales were set on basis of ROC curve analysis where sensitivity and specificity curves cross on the graph.

To identify the combination of scales and items with the most precise predictive value for CG, we performed a multivariate model analysis. Sensitivity, specificity and AUC were calculated for the most promising models. The final model was selected to possess a high predictive performance in detecting risk of CG based on the best possible balance between sensitivity and being brief. Finally, the model was transformed into a questionnaire for use in the clinic.

## Results

Participants differed from non-participants in terms of age (participants: 67 (SD = 11.75), non-participants: 73 (SD = 7.3), p < 0.001) and gender (participants: 60% females, non-participants: 74% females, p < 0.001). A BDI score of 10 or more was obtained at baseline by 35% of the participants, who also answered the questionnaire at T2 while 45% of the participants, who only replied at baseline, scored 10 or more on the BDI at T1 (p = 0.082). Cronbach's α for the ICG-R in this study was 0.90. Participant characteristics are shown in Table 1 in terms of gender and age and their score on the ICG-R, the BDI and the single item C.

**Table 1:** Descriptive characteristics of participants.

	All		ICG >42		BDI >9		Item C >4	
	n	%	n	%	n	%	n	%
**All**	276	100	54	19.6	98	35.5	39	14.1
								
**Population**								
Palliative sample	74	26.8	16	21.6	36	48.6	11	14.9
Cpr sample	202	73.2	38	18.8	62	30.7	28	13.9
**Gender**								
Female	165	59.8	37	22.4	65	39.4	18	10.9
Male	111	40.2	17	15.3	33	29.7	21	18.9
**Age**								
15-65	62	22.5	12	19.4	30	48.4	8	12.9
66-69	65	23.6	13	20.0	20	30.8	7	10.8
70-73	71	25.7	13	18.3	23	32.4	9	12.7
74-83	78	28.3	16	20.5	25	32.1	15	19.2

ICG-R = Inventory of Complicated Grief - Revised.

BDI = Beck's Depression Inventory.

Item C = "Even while my relative was dying, I felt a sense of purpose in my life".

ROC curve analysis was performed to seek predictive variables. The initial ROC curve analyses are shown in Table 2.

**Table 2:** ROC curve analysis on all scales and items on the dataset.

Variable	AUC	95% CI	
Gender	0.447	0.380	0.513
Age	0.508	0.424	0.591
Education	0.479	0.380	0.579
No of children	0.422	0.344	0.500
**Item A**	**0.712**	**0.644**	**0.780**
**Item B**	**0.707**	**0.623**	**0.791**
**Item C**	**0.730**	**0.656**	**0.805**
**BDI**	**0.827**	**0.767**	**0.888**
**HTQ (16)**	**0.821**	**0.758**	**0.884**
CSS	0.458	0.373	0.542
CSQ rational	0.528	0.448	0.609
**CSQ emotional**	**0.753**	**0.684**	**0.821**
CSQ detached	0.419	0.342	0.497
CSQ avoidance	0.591	0.508	0.675
SOC	0.347	0.261	0.433
SWLS	0.341	0.261	0.421
NEO Conscient	0.457	0.372	0.542
NEO Altruism	0.493	0.402	0.584
NEO Openness	0.497	0.412	0.582
**NEO Neurotic**	**0.667**	**0.587**	**0.748**
NEO Extrovert	0.468	0.376	0.560

*The area under the curve (AUC) was measured against the score of the Inventory of complicated Grief-Revised (ICG-R). A score of >= 43 six months after bereavement indicated complicated grief. Only variables with AUC>0.65 were considered for further analysis*.

Item A, B and C: Single item questions on distress experienced in relation to the death of their relative (see text); BDI: Beck's Depression Inventory; HTQ: Harvard Trauma Questionnaire; CSS: Crisis Support Scale; CSQ: Coping Style Questionnaire (also tested based on its four subscales); SOC: Sense of Coherence; SWLS: Satisfaction with life scale; NEO: NEO Personality Inventory (tested based on the subscales).

BDI was the scale with the highest AUC (AUC = 0.83) and thereby was chosen for further analysis over HTQ, the emotional subscale of CSQ and the neuroticism subscale of NEO-PI-R. The choice of the BDI was based on the fact that a brief model was given high priority and the BDI is a full scale which is well validated in various populations. Correlation between the full scale BDI and the ICG-R with a cut off point of 43 was 0.48. The optimal cut off point on the BDI for the purpose of prediction turned out to be 10. Gender, age, education and number of children all showed an AUC<0.51 and weren't chosen for further analysis. The single item questions A, B and C all had an AUC >0.70 and were eligible for the multivariate model analysis.

Model 3 with the BDI and the single item C yielded a sensitivity of 0.796, a specificity of 0.752 and an AUC = 0.81 and was chosen as the model with the best predictive performance. This model was converted into a clinical tool where the BDI scores and item C could be translated into three risk categories:

Risk group 1: a BDI score of 0-9 and item C score of 1-4

Risk group 2: a BDI score of 10-19 or a BDI score of 0-9 and item C score of 5-7

Risk group 3: a BDI score of 20-63 or a BDI score of 10-19 and item C score of 5-7

This model allowed the detection of 46 (85.2%) of 54 bereaved patients with complicated grief, defined by a score of 10 or above on the BDI or a score of 5 or above on the Item C (sensitivity = 0.852, specificity was 0.694, with positive predictive value (PPV) of 40.4% and negative predictive value (NPV) of 95.1%). Specificity was improved to 0.964 with a score of 20 or above on the BDI or a score of 10 or above on the BDI and a score of 5 or above on the Item C at the expense of sensitivity (0.407).

## Discussion

### Principal findings

In this study it was possible to identify a combination of the BDI scale and a single item (*Even while my relative was dying, I felt a sense of purpose in my life) *answered at eight weeks post loss to assess the propensity of bereaved individuals to develop complicated and prolonged reaction of grief after six months. Hence, we were able to construct a screening tool to identify people at risk of suffering complicated grief six months after bereavement and divide the risk of a pathological grief reaction of bereaved individuals into three distinct groups. This study points to the necessity of awareness of a depressive symptomatology among older people and family caregivers to deceased cancer patients in connection with bereavement as it might predict complications in the process of grief reactions.

### Strengths and weaknesses

The sample size of this study was acceptable but a larger sample may have added more statistical precision to the estimates. Though the sample in this study was population-based, there was a drop-out. Furthermore, part of this sample was recruited through a palliative care team, which means there is a risk of selection bias that need to be taken into account when considering the representativity of this population. Analyses showed that older people and females were underrepresented in this sample yet overall the mean age of the population in the sample was relatively high. However, this means that the results might be underestimating the risk for older people and females, which should be taken into consideration when applying the screening tool and cut points might need adjustment in future studies.

Another weakness that needs to be touched upon is the limitation in the performance of the screening tool. It was possible to identify a screening tool for early identification of individuals at risk of developing complicated grief, yet the tool seems to have some shortcomings that need to be taken into consideration when applying it in a clinical setting. The PPV of the potential screen was 40% for risk group 2 and the PPV for risk group 3 was 73% and therefore, we recommend to use only the cut off for risk group 3 in clinical practice when applied in addition to the clinical judgment of the professional.

A notable methodological weakness in this study and generally in studies on bereavement is the lack of a clear and distinct diagnosis and measure of pathological grief, which makes conclusions ambiguous to information bias and the lack of criterion validity. The ICG-R is a widely used self-report questionnaire on CG but still lacks research in validation of cut off points and in non-American populations. In this study we had to define a usable clinical cut off point, as the ICG-R is not standardised in a Danish population. We chose to define the cut off point of the ICG-R based on a syndromal level, inspired by the authors of the original study to avoid over-estimating the risk of CG. Future studies will be helpful in estimating and validating cut off points for the ICG-R as well as the criterion validity for the detection of pathological grief reactions.

The issue regarding generalizability revolves around two issues; will the model apply and perform reasonably or better in other populations and in other settings and can the association between the BDI and the ICG-R also be found in other groups. Further research is needed to elucidate the screening properties in other settings and in other populations, especially to younger individuals and to populations with sudden and unexpected losses. However we estimate, that the association found between the screening tool and the risk of complicated grief can be generalised to other populations.

This study does not directly address the issues on CG or PGD as a conceptual and diagnostic entity and the present tool is not developed as a diagnostic scale but rather a prognostic tool. It should be addressed though, that a depression inventory in this study turns out to be capable of predicting CG in spite of CG has proven to be a symptom cluster different than depression. Already when developing the ICG, the authors noted the high association between the BDI and the ICG. One of the reasons for this is probably the high correlation between clusters of symptoms, which makes the syndromes hard to differentiate from one another in the clinic. Another point that needs to be made in regard to the association between depression and complicated grief is the population in this study, which consisted mainly of older people and family caregivers to deceased cancer patients receiving palliative care. In this population one might assume, that depression is a better predictor of CG than for instance PTSD while it could be hypothesized that PTSD might be a better predictor in populations with more sudden or unexpected losses.

Earlier findings have focused on the assessment of symptoms of complicated grief. As a supplement to that, this study found, that it is possible with screening to early identify bereaved individuals, who might be at risk of developing complications following bereavement. The implications of an effective screening tool could be to aid the clinician make evidence-based clinical decisions and channel resources into targeted early intervention strategies, decrease the frequency of cases with prolonged reactions and prevent unnecessary suffering. However, the screening tool presented needs validation in a clinical setting to prove its validity and applicability to clinical work and in other populations. Furthermore, a shorter version of a screening tool is to be wished for to improve acceptability and response rates, when used in clinical settings. More research is needed to continue the process of successful grief screening.

## Conclusion

This study showed that the BDI in combination with a single item question eight weeks post loss may be used for clinical screening for bereaved individuals at risk of developing prolonged complicated grief. Further validation will be needed to consider this screening tool for clinical work. A screening tool can be crucial in the identification of bereaved individuals susceptible to developing complications during a period of grief. Early identification of individuals at risk of developing CG will be helpful in combination with the clinical assessment in the allocation of resources and provision of targeted support to the bereaved in general practice, in palliative care or elsewhere in the health care systems.

## Abbreviations

AUC: Area under the curve; BDI: Beck's Depression Inventory; CG: Complicated Grief; CSS: Crisis Support Scale; CSQ: Coping Style Questionnaire; CPR: Central Person Registry; HTQ: Harvard Trauma Questionnaire; ICG-R: Inventory of Complicated Grief-Revised; NEO-PI-R: NEO Personality Inventory -Revised; NPV: Negative Predictive Value; PGD: Prolonged Grief Disorder; PPV: Positive Predictive value; PTSD: Posttraumatic Stress Disorder; ROC: Receiver operating Curve; SOC: Sense of Coherence; SWLS: Satisfaction with Life Scale

## Competing interests

The authors declare that they have no competing interests.

## Authors' contributions

MG carried out some of the data collection, conceived of the study and drafted the manuscript. MO inspired the design of the study, carried out most of the data collection and participated in the critical revision of the manuscript. IS participated in the design of the study and performed the statistical analysis. ABJ supervised the study and participated in critical revision of the manuscript. PV supervised the study, helped perform statistical analysis and participated in critical revision of the manuscript. All authors read and approved the final manuscript.

**Table 3:** The multivariate model analysis with the BDI and single items with sensitivity (SE), specificity (SP), positive predictive value (PPV) and negative predictive value (NPV)with 95% confidence intervals (CI) and areal under the curve (AUC)

Model	N	SE (%)	SP (%)	PPV (%)	NPV (%)	AUC
		(95% CI)	(95% CI)	(95% CI)	(95% CI)	
**Model 1**						
BDI, cut10	268	78.0 (64.0-88.5)	75.7 (69.4-81.2)	42.4 (32.2-53.1)	93.8 (89.1-96.8)	0.82
Item A, cut5						
						
**Model 2**						
BDI, cut10	261	72.9 (58.2-84.7)	79.8 (73.8-85.0)	44.9 (33.6-56.6)	92.9 (88.-96.16)	0.84
Item A, cut5						
Item B, cut5						
						
**Model 3**						
BDI, cut10	276	79.6 (66.5 - 89.4)	75.2 (69.0 - 80.8)	43.9 (33.9 - 54.3)	93.8 (89.2 - 96.9)	0.81
Item C, cut5						
						
**Model 4**						
BDI, cut10	266	70.0 (55.4-82.1)	79.6 (73.6-84.8)	44.3 (33.1-55.9)	92.0 (87.1-95.4)	0.84
Item A, cut5						
Item C, cut5						

**Table 4:** The final model: The three risk groups with sensitivity (SE), specificity (SP), positive predictive value (PPV) and negative predictive value (NPV) with 95% confidence intervals (CI) and prevalence of complicated grief (ICG-R).

Risk group	N (%)	SE (%)	SP(%)	PPV (%)	NPV (%)	ICG-R (%)
		(95% CI)	(95% CI)	(95% CI)	(95% CI)	
**1**	162 (58.7)	100	0			5
**2**	84 (30.4)	85.2 (72.9-93.4)	69.4 (62.9-75.4)	40.4 (31.3-49.9)	95.1 (90.5-97.8)	29
**3**	30 (10.9)	40.7 (27.6-55.0)	96.4 (93.0-98.4)	73.3 (54.1-87.7)	87.0 (82.1-90.9)	73

N = 276.

The risk groups:

Risk group 1: a BDI score of 0-9 and item C score of 1-4;

Risk group 2: a BDI score of 10-19 or a BDI score of 0-9 and item C score of 5-7;

Risk group 3: a BDI score of 20-63 or a BDI score of 10-19 and item C score of 5-7

## Pre-publication history

The pre-publication history for this paper can be accessed here:

http://www.biomedcentral.com/1472-684X/10/9/prepub
